# Synergistic mechanisms of humic acid and biomineralization in cadmium remediation using *Lysinibacillus fusiformis*

**DOI:** 10.1111/1758-2229.70037

**Published:** 2024-11-13

**Authors:** Wei Zhou, Yaqi Zhu, Varenyam Achal

**Affiliations:** ^1^ Department of Environmental Science and Engineering Guangdong Technion‐Israel Institute of Technology Shantou China; ^2^ Guangdong Provincial Key Laboratory of Materials and Technologies for Energy Conversion Guangdong Technion‐Israel Institute of Technology Shantou China

## Abstract

Heavy metal pollution, particularly cadmium, poses severe environmental and health risks due to its high toxicity and mobility, necessitating effective remediation strategies. While both microbially induced carbonate precipitation (MICP) and humic acid adsorption are promising methods for heavy metal mitigation, their combined effects, particularly the influence of humic acid on the MICP process, have not been thoroughly investigated. This study explores the interaction between humic acid and MICP, revealing that humic acid significantly inhibits the MICP process by reducing urease activity, with the 10% humic acid treatment resulting in a 23.8% reduction in urease activity compared to the control. Additionally, while higher concentrations of humic acid did not significantly reduce cadmium ion concentrations, they did result in a slight increase in organically bound cadmium, indicating an interaction that could alter metal speciation in the soil. These findings provide important insights into the mechanisms by which humic acid affects MICP, offering a foundation for optimizing combined remediation approaches. Future research should aim to fine‐tune the balance between MICP and humic acid to enhance the overall efficiency of cadmium remediation strategies. This study contributes to the development of more effective and sustainable methods for addressing cadmium contamination.

## Graphical abstract

Humic acid at high concentrations impacts microbially induced carbonate precipitation (MICP) by inhibiting urease activity, reducing carbonate production and increasing organically bound cadmium, affecting cadmium immobilization efficiency. This study uniquely explores the combined effects of MICP and humic acid adsorption on cadmium remediation, revealing the inhibitory impact of humic acid on urease activity and MICP efficiency. These findings highlight the complex interplay between organic matter and biomineralization, paving the way for optimized remediation strategies.

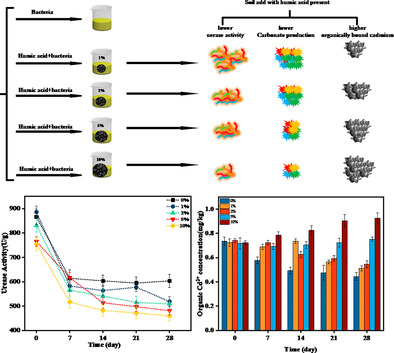

## INTRODUCTION

Environmental and public health concerns arise from the global issues of heavy metal toxicity and bio‐accumulation. The modernization of various industries, urbanization and agriculture practices has led to severe damage to ecosystems and lands in both developed and developing nations (Sahab et al., 2021; Suhani et al., 2021). The contamination of soil and water with heavy metals poses a significant threat to human health and the environment. Among the heavy metals, cadmium (Cd) toxicity is a significant concern in countries such as China, Bangladesh, India and Pakistan (Du et al., 2020; Latif et al., 2020; Saini & Dhania, 2020; Shahriar et al., 2020). Currently ranked seventh on the list provided by the Agency for Toxic Substances and Disease Registry (ATSDR) (Latif et al., 2020; Singh et al., 2021), Cd can mimic other important divalent metals, playing crucial roles in several biological processes in nature. While traditionally no biological function for Cd in terrestrial plants or animals has been documented (Saini & Dhania, 2020), research suggests that in marine environments, Cd can serve as the active site of carbonic anhydrase in diatoms, facilitating their carbon acquisition process (Xu et al., 2008). Despite this specific role, Cd is generally recognized as an eco‐toxic heavy metal that adversely affects various biological processes in plants, animals and humans.

In China, Cd carries the dubious distinction of being the most prevalent metal pollutant. This is because of its high toxicity and mobility and the fact that it is concentrated in more areas than other metals (Ye et al., 2015). The average Cd content in agricultural soils in most Chinese cities is 0.43 mg/kg (Wei & Yang, 2010). However, the maximum permissible concentration of potentially hazardous elements (PTE‐MPC) for Cd in Chinese agricultural soils is 0.3 mg/kg (Wei & Yang, 2010). Interestingly, this limit is significantly higher than the country's background Cd level.

The condition of Cd‐enriched farmland soil in China and other countries is concerning, and effective remediation measures are urgently needed to address the shortcomings of current methods (Achal et al., 2012; Tang et al., 2016). Microbially induced carbonate precipitation (MICP), a biomineralization process, is a naturally occurring biochemical process found in soil, water and a variety of ecosystems including fields, caves, freshwater, marine sediments and hypersaline habitats (Castro‐Alonso et al., 2019). Prominent soil bacteria such as *Sporosarcina*, *Bacillus*, *Shewanella* and *Exiguobacterium* play a vital role in facilitating this process. Enzymes such as urease, carbonic anhydrase and asparaginase initiate the process, with urease being the most commonly utilized and energy‐efficient method for carbonate production. Urea holds immense value as a nitrogen source for various organisms (Liu et al., 2021).

The MICP process encompasses three distinct stages: carbonate generation, crystal formation and mineral precipitation (Jalilvand et al., 2020). Within this process, either native or introduced microorganisms produce urease, which catalyses the hydrolysis of urea (NH_2_CONH_2_) into ammonia (NH_3_) and carbon dioxide (CO_2_), resulting in the formation of ammonium ions (${\mathrm{NH}}_{4}^{+}$) and hydroxide ions (OH—). This reaction leads to an increase in the surrounding pH and provides a nucleation site for promoting the precipitation of carbonate (${\mathrm{CO}}_{3}^{2-}$) in a calcium‐rich environment (Rong et al., 2020). In an alkaline environment, the bicarbonate ions (${\mathrm{HCO}}_{3}^{-}$) interact with the hydroxyl ions (OH^−^) to produce carbonate ions (${\mathrm{CO}}_{3}^{2-}$). The presence of calcium ions (Ca^2+^) triggers the formation of calcium carbonate (CaCO_3_), which precipitates due to its low solubility in water. The reactions involved in the process are as follows (Cardoso et al., 2020):

CO(NH_2_)_2_ + H_2_O → 2NH_3_ + CO_2_.

2NH_3_ + 2H_2_O ↔ 2${\mathrm{NH}}_{4}^{+}$ + 2OH^−^.

CO_2_ + H_2_O ↔ ${\mathrm{HCO}}_{3}^{-}$ + H^+^.

${\mathrm{HCO}}_{3}^{-}$ + H^+^ + 2OH^−^ ↔ ${\mathrm{CO}}_{3}^{2-}$ + 2H_2_O.

Ca^2+^ + ${\mathrm{CO}}_{3}^{2-}$ ↔ CaCO_3_↓.

The MICP technique has been demonstrated to be efficacious in the removal of heavy metals from contaminated soils on several occasions (Sharma et al., 2022); nevertheless, although MICP is environmentally friendly and sustainable, its implementation is influenced by multiple factors such as bacterial species, soil pH, water content and soil composition. Among these factors, humic acid is a key determinant (Tang et al., 2020). Humic acid (HA) constitutes a crucial organic component of soils and is also the most chemically active and unstable element of the humic substrate. Its unique properties have been the subject of extensive research, resulting in a diverse range of applications in various fields (Rashad et al., 2022).

In recent times, there has been significant attention and interest in the interaction between HA and heavy metals. Therefore, it is essential to examine the effects of humic substances when applying MICP to organic soils. This is because humic substances can inhibit urease activity and disrupt the formation of calcium carbonate. Specifically, the presence of HA in organic soils is a major concern. HA is a widely used soil fertilizer because of its excellent cation exchange capacity and ability to hinder soil urease activity. It can simultaneously impact both heavy metals and the MICP process. On one hand, it can decrease the mobility and toxicity of heavy metals, effectively reducing their bioactivity in soil. On the other hand, it can inhibit the growth of soil bacteria and urease activity. HA blocks the enzyme's active site, affecting urea hydrolysis efficiency. Additionally, during the MICP process, humic acid can interfere with the formation of CaCO_3_ by interacting with calcium ions (Chen et al., 2023). Therefore, our study aims to evaluate the synthesis impact of humic acid on the precipitation of cadmium by microbes. Limited literature explores the effects of humic acid on heavy metals and the MICP process. Our research will provide a more comprehensive understanding of heavy metal precipitation and purification, the mechanisms and challenges of MICP processes and the role of humic acids in these processes. Ultimately, this study will contribute to the establishment of a strong foundation for sustainable environmental development.

## EXPERIMENTAL PROCEDURES

### Preparation of contaminated soil samples

To simulate a heavy metal‐contaminated environment, we utilized a mixture of nutrient broth containing urea (referred to as NBU, composed of nutrient broth with 2% urea by weight) as our growth media. Cadmium chloride (CdCl_2_, analytical reagent grade) and humic acid (HA, analytical reagent grade) were incorporated to prepare standard contaminated soil samples. This setup was designed to support the growth and activity of the bacterium *Lysinibacillus fusiformis* (Fang et al., 2021), used in the present study for MICP.

Soil samples were collected from the foothills of the Guangdong Technion Campus in Shantou, China, specifically from the upper 20 cm of the soil profile. Post‐collection, these samples were oven‐dried, manually cleared of organic debris such as roots and leaves and sieved through a 2 mm mesh to ensure uniformity. The physical and chemical characteristics of the soil were analysed to establish a baseline for further experimentation. For the cadmium remediation trials, we established experimental plots in a controlled environment room maintained between 25 and 30°C. Each plot consisted of 200 g of sieved soil artificially spiked with cadmium to achieve a final concentration of 5 mg/kg. We varied the humic acid content across different plots at concentrations of 1%, 2%, 5% and 10%, aiming to assess its impact on cadmium mobility and microbial remediation efficiency. The soil was then augmented with NBU media supplemented with a calcium source to promote the MICP process.

### Experimental design and process

Our experimental design included multiple treatment groups, each receiving different concentrations of humic acid, as outlined previously, alongside a cadmium concentration of 5 mg/kg, in the form of CdCl_2_. Each plot was saturated with the NBU media to ensure optimal microbial activity; a control group was maintained under similar conditions but without cadmium addition.

Over a one‐month period, we followed a rigorous sampling schedule to monitor the progress of cadmium remediation. Soil samples were collected at 7‐day intervals for immediate chemical analysis. This systematic collection was crucial for assessing the efficacy of the MICP process and the role of humic acid in enhancing cadmium immobilization. The entire experimental sequence was conducted under consistent conditions to maintain the integrity of the results.

### Assessment of soil urease activity

Soil urease activity was assessed using the sodium phenol‐sodium hypochlorite colorimetric method, facilitated by the Soil Urease (S‐UE) Activity Detection Kit provided by Sangon Biotech. This assay measures the ammonia liberated from urea, which serves as an indicator of urease activity in soil samples.

Initially, 0.25 g of oven‐dried soil was accurately weighed and moistened with 125 μL of Reagent I. The soil was mixed until fully saturated and allowed to stand at room temperature for 15 min to enable the urease in the soil to initiate the reaction. Subsequently, 625 μL of Reagent II was added to the sample, while in control samples, Reagent II was substituted with an equal volume of distilled water. An additional 1250 μL of Reagent III was introduced to the mixture, which was then thoroughly mixed and incubated in a 37°C water bath for 24 h. Post‐incubation, the samples were centrifuged at 10,000 g for 10 min at room temperature, and the supernatant was collected.

To ensure appropriate assay sensitivity, the supernatant was diluted tenfold (0.1 mL of supernatant in 0.9 mL of distilled water). If the initial absorbance reading exceeded a value of 1, further dilution was performed. For colour development, 360 μL of the diluted supernatant was combined with 120 μL each of Reagents IV and V. The mixture was thoroughly mixed and left to stand at room temperature for 20 min. Following this, 400 μL of distilled water was added prior to zeroing the spectrophotometer at 630 nm for absorbance measurement.

The difference in absorbance between the measurement and the reference tubes was recorded as ∆A. To calibrate the results, a standard curve was generated using known concentrations of a nitrogen standard solution (ranging from 0 to 8 μg/mL). The absorbance for each standard concentration was measured, subtracting the value of the blank (0 μg/mL) to correct for baseline fluctuations. This standard curve facilitated the quantification of urease activity in the soil samples, providing insights into the enzymatic activity influenced by treatment conditions within the experimental framework.

The measurement of enzyme activity is established by the generation of 1 μg NH_3_‐N per g of soil sample within a day.

Urease activity (U/g) = X*10*V_reverse total_/W/T = 80*X.

**10:** Dilution ratio;

**T:** Reaction time, 1 day;

**V**_**reverse total**_: total volume of reaction system: 2 mL;

**W:** Sample mass, 0.25 g.

### Cadmium analysis

To determine the cadmium concentration in soil samples, a sequential chemical extraction method developed by McGrath and Cegarra (1992) was employed, targeting various forms of cadmium. The procedure encompassed four distinct phases:

- Exchangeable Cadmium Extraction: Initially, soil samples were collected and left to equilibrate for 5 days. Subsequently, 3 g of each sample were placed into 30 mL of a 1 M solution of CaCl_2_. These samples were agitated for 16 h and then centrifuged at 13,200 g for 10 min, separating the supernatants that contained the exchangeable cadmium.
- Organically Bonded Cadmium Extraction: The residues from the previous step were then subjected to further extraction. Each residue was shaken for 16 h with 30 mL of a 0.5 M NaOH solution. After centrifugation, the supernatants were collected and treated with aqua regia (a 4:1 v/v mixture of 65% nitric acid and hydrochloric acid) to extract cadmium bonded to organic matter.
- Carbonate‐Bonded Cadmium Extraction: Following the extraction of organically bonded cadmium, the second set of residues underwent an additional shaking process with 30 mL of a 0.05 M EDTA‐2Na solution for 16 h. After separation by centrifugation, these supernatants were also treated with aqua regia to target cadmium associated with carbonates.
- Residual Cadmium Extraction: The final residues were digested in aqua regia to extract any remaining cadmium. One millilitre of the digested solution was filtered through a 0.45 μm membrane filter, and the filtrate was diluted with 9 mL of 2% nitric acid to adjust the pH. The mass of all samples from steps a, b and c, both with and without supernatant, was carefully recorded to ensure comprehensive data collection.

After appropriate dilution of the samples using 2% nitric acid, heavy metal measurements were performed using an Inductively coupled plasma⸺optical emission spectrometry (ICP‐OES) system (Thermo Scientific iCAP7000 Plus Series ICP‐OES) calibrated with heavy metal standards within the detection range.

### Scanning electron microscopy with energy dispersive spectroscopy (SEM‐EDS)

Prior to conducting SEM analysis, a set of pretreatment techniques were applied to the soil samples. Initially, the samples were subjected to a drying process in an oven set at 65°C for a duration of 24 h. Subsequently, they were sealed and preserved at a temperature of 4°C. To investigate the morphology and microstructure of the precipitated carbonates, a gold sputter coating was administered to the samples before being analysed using a ZEISS Gemini450 scanning electron microscope with energy dispersive spectroscopy (EDS).

### X‐ray diffraction (XRD) analysis

In this study, we employed Rigaku SmartLab® high‐resolution X‐ray diffractometer to thoroughly examine soil samples taken following the remediation process. After naturally drying, the samples were ground with a glass mortar. We utilized a Bruker D8 diffractometer with 40 kV and 30 mA, scanning from 5 to 80° in steps of 0.02° to obtain XRD spectra. This allowed us to accurately identify biominerals and other sediments present in the soil.

### Carbonate production by thermogravimetric/differential thermal analysis (TG‐DTA)

Thermogravimetric/differential thermal analysis (TG‐DTA) was used to quantify the carbonate content produced following soil remediation across five different treatment scenarios. We conducted thermal analysis on CaCO_3_, CdCO_3_ powders and mixtures of both using a thermo‐mechanical analysis equipment (Shimadzu Simultaneous DTA‐TG DTG‐60A).

For each analysis, a 5 mg sample was precisely weighed and placed in an alumina pan. The samples were then subjected to a controlled heating schedule, where they were gradually heated to a maximum temperature of 900°C at a rate of 50°C per min. Upon reaching the peak temperature, the samples were held at 900°C for a 10‐min heat‐soak period. During the heating process, we continuously monitored the sample weight and recorded any thermal events, such as exothermic or endothermic reactions, to assess the stability and decomposition patterns of the carbonates formed during soil remediation.

## RESULTS AND DISCUSSION

### Urease activity in soil samples

The experiment involved five groups of soil samples containing Cd and treated with different concentrations of humic acid (0%, 1%, 2%, 5% and 10%) along with nutrient broth with urea (NBU) and a blank control without urea (Blank). Soil samples were collected at intervals of 7 days following the termination of the artificial experiment (Zhu et al., 2016).

**FIGURE 1:**
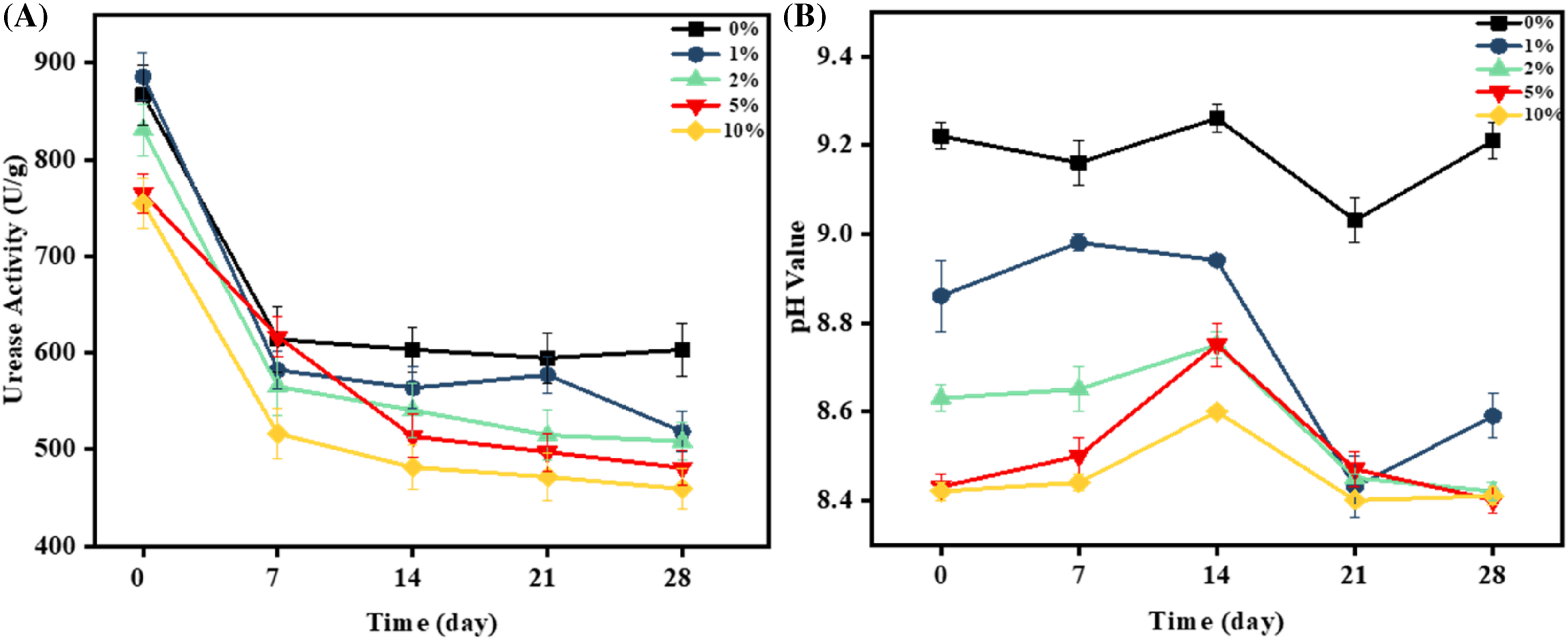
Comparative analysis of soil treatments: (A) Urease activity and (B) pH values in soil supplemented with 5 mg Cd/kg soil across varying concentrations (0%–10%) of humic acid treatments. Each data point in both (A) and (B) represents the mean of three replicates (*n* = 3), with error bars depicting standard deviation.

Among all the treatments, the soil samples treated with humic acid showed lower urease activity as the concentration of humic acid increased (Figure 1). Li et al. (2022) investigated that the humic acid can inhibit the nitrogen removal rate by effecting the production and the activity of nitrifying and denitrifying enzymes. It is observed in Figure 1A that the initial soil urease activity was significantly higher due to the saturation of the soil sample caused by added bacterial cells during cultivation. From the seventh day onwards, the MICP process continued after sampling and the addition of the bacterial solution.

Throughout the entire process, the urease activity of the soil showed a decreasing trend, and it demonstrated an increase with higher concentrations of humic acid. The trend in soil urease activity was as follows: 0% > 1% > 2% > 5% > 10%. The group with a 10% humic acid content exhibited 23.8% lesser urease activity compared to the group without humic acid, indicating that the presence of humic acid has a certain inhibitory effect on bacterial urease production, The findings align with other report (Li, Koopal, et al., 2022), indicating that the inclusion of humic acid extraction can effectively impede urease activity, leading to an extended release period of ammonia nitrogen, which cannot be reversed. Further, Liu et al. (2019) have also shown that the addition of humic acid has a considerable impact on enzymatic stability and activity.

Further, we also tested the influence of HA on the pH levels of soil treated under conditions conducive to MICP. Our experiments tracked soil pH over a 28‐day period with the application of varying concentrations of HA (0%, 1%, 2%, 4%, 5% and 10%). The results, illustrated in Figure 1B, demonstrated a slight but consistent decrease in pH across all tested concentrations. Notably, despite this decrease, the pH remained well within the optimal growth range for *Lysinibacillus fusiformis*, a key bacterium in MICP processes.

The experimental data presented substantiates the efficacy of humic acid in MICP process, confirming that its mild acidifying effects do not adversely affect the microbial activities essential for effective bioremediation. These results endorse the integration of humic acid into MICP treatments, where it serves a dual role; it enhances microbial efficacy while only minimally altering soil chemical properties. Therefore, humic acid emerges as a beneficial amendment for the bioremediation of heavy metal‐contaminated soils, supporting microbial processes without imposing harmful stress on the microbial communities involved. This balance highlights its potential as a versatile agent in environmental remediation strategies.

### Cadmium concentration in different soil samples

**FIGURE 2:**
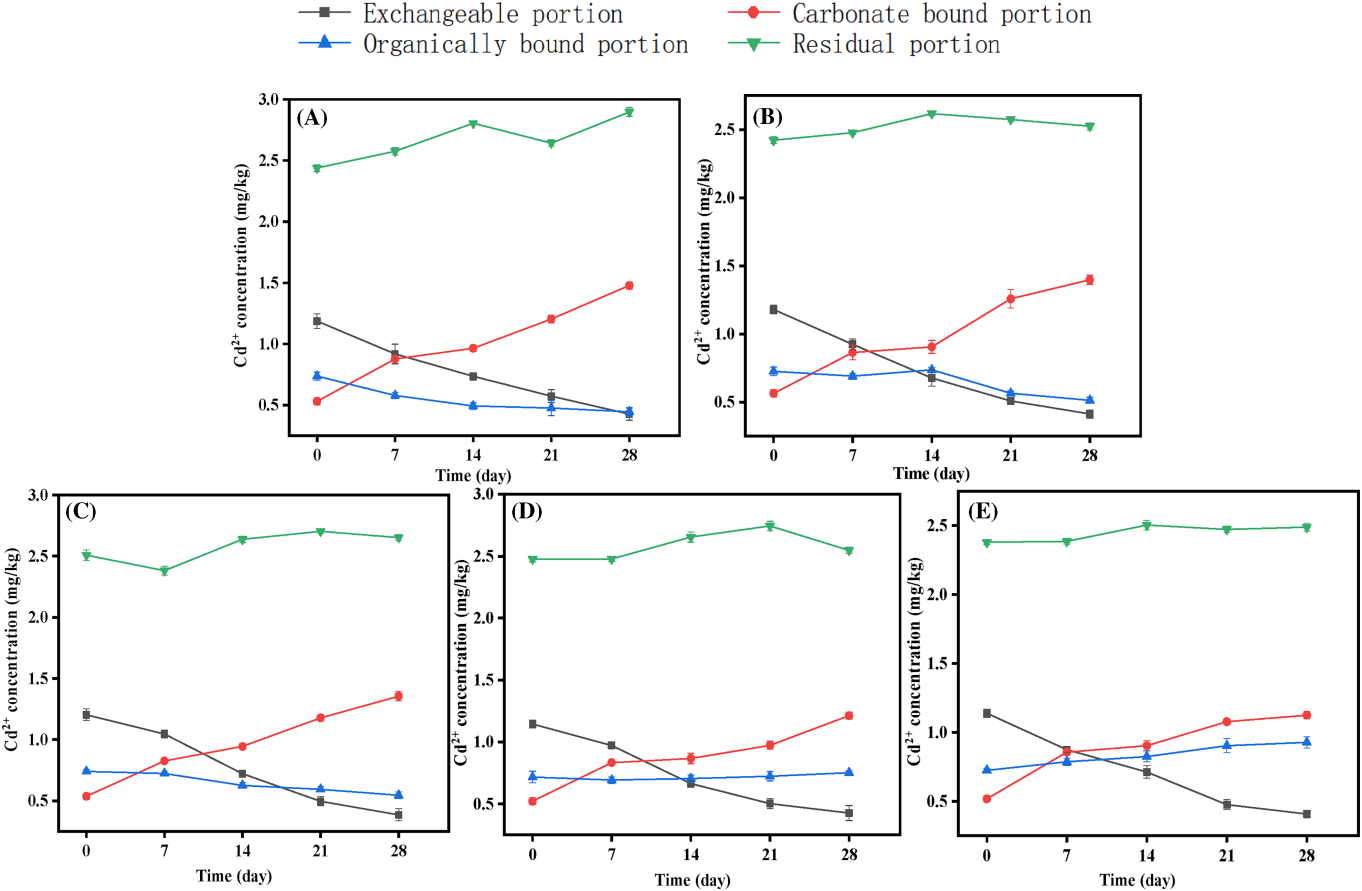
The distribution of cadmium in different fractions of soils in different treatments (A–E represent 0%, 1%, 2%, 5% and 10% of humic acid‐treated sample).

A sequential extraction procedure was employed to analyse the distribution of cadmium in different soil fractions and investigate the interaction of Cd with biominerals through carbonate precipitation processes in contaminated soils. The four forms of heavy metals considered in this study were exchangeable, carbonate‐bound, organically bound and residual. The content of cadmium in the soil samples is presented in Figure 2.

Overall, all four forms of cadmium display distinct patterns, with the residual form being relatively stable. The figures indicate a general decreasing trend for exchangeable metal ions, even in the blank group (without bacterial solution), where the exchangeable cadmium ions decreased by 13.1% after 28 days, whereas the group without humic acid experienced a more substantial decline of 64.1%. In contrast, groups treated with varying levels of humic acid (1%, 2%, 5%, 10%) displayed a tiered reduction in exchangeable cadmium: 65.1%, 68%, 62.7% and 64.2%, respectively. Similar observation was found by Wu et al. (2017), where there was a notable decrease in the concentration of available heavy metal portions, particularly for Cu and Pb, as the concentration of humic acid rises.

Moreover, the overall trend for cadmium ions in the carbonate state showed an increase. While cadmium ions in the carbonate state remained relatively unchanged or slightly increased in the blank group, there was a significant increase observed in the experimental group. The research outcomes revealed that the concentration of cadmium ions decreased as humic acid content increased. Notably, the highest increase in carbonate‐bound cadmium (152%) was recorded for samples without humic acid, whereas the lowest increase (83.5%) was observed for samples with 10% humic acid. Samples containing 1%, 2% and 5% humic acid showed increases of 127%, 96.8% and 87.2% in carbonate‐bound cadmium, respectively.

These findings closely mirror the results of experiments conducted by other researchers (Zhao et al., 2022), who reported that the use of Si fertilizer and humic acid (HA) significantly impact soil attributes such as pH, electrical conductivity, total organic carbon, water‐soluble organic carbon and nitrate‐nitrogen. The incorporation of HA and Si‐HA (silicon‐humic acid) led to a marked reduction in the availability of Cu, Cd, Pb and Zn. Additionally, no significant variation was observed in the physicochemical makeup of soil and soil aggregates. However, the introduction of HA and Si‐HA considerably reduced the availability of Cu, Cd, Pb and Zn in soil aggregates, with concentrations higher in microaggregates than in macroaggregates. The inclusion of these compounds altered the levels of heavy metals in different particle sizes, eventually transforming them into the residual state.

This indicates that with continuous supplementation of culture medium and bacterial solution, the MICP process continues to play an active role. However, in the experimental groups containing humic acid, the rate of increase in carbonate‐bound cadmium ions slowed as humic acid content increased, suggesting that humic acid presents certain obstacles to carbonate formation. Overall, cadmium ions exhibited a decreasing trend, but as humic acid concentration increased, there was also a modest rise in organically bound cadmium ions, indicating that the organic components in humic acid have a certain adsorption effect on metal ions. This comprehensive analysis underscores the nuanced interplay between humic acid concentrations and heavy metal bioavailability, highlighting potential of HA to modify soil chemistry and its implications for heavy metal management in contaminated environments.

### Microstructure of soil samples

**FIGURE 3:**
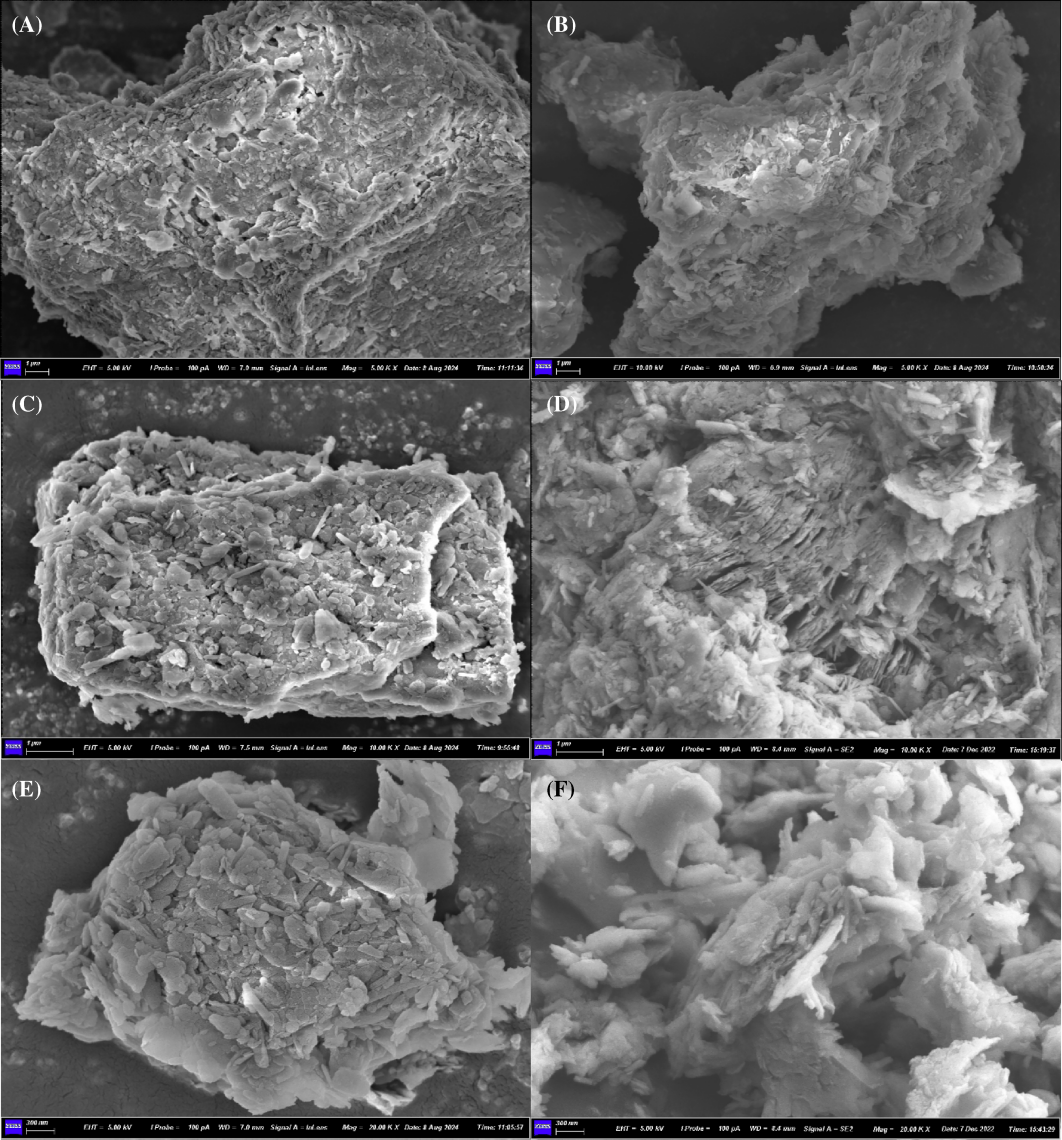
Scanning electron microscopy images showing the morphological changes in cadmium‐contaminated soils treated with varying concentrations of humic acid. (A, B) Soil samples treated with 1% humic acid for 14 days without bacterial inoculation, exhibiting rough surfaces with minimal calcite formation. (C, D) Samples treated with 2% and 5% humic acid for 14 days, displaying smaller, more compact calcite crystals embedded in a delicate matrix. (E, F) Soil samples treated with 1% and 10% humic acid for 28 days, revealing further reductions in calcite crystal size and smoother surfaces as humic acid concentration increases. The images illustrate the impact of humic acid concentration on the size, distribution and morphology of carbonate‐bound cadmium precipitates in the soil, highlighting the inhibitory effect of higher humic acid levels on crystal growth.

The SEM images (Figure 3) provided a detailed view of the morphological changes in cadmium‐contaminated soils treated with varying concentrations of humic acid, both with and without bacterial inoculation, over 14 and 28 days. The micrographs reveal distinct differences in the texture and mineral composition of the soil samples subjected to different treatments.

In the soil samples treated with 1% humic acid and incubated for 14 days without bacterial inoculation (Figure 3A,B), the surface appears rough, with less evident calcite formation, indicating minimal microbial activity and carbonate precipitation. This is consistent with the observed roughness and lack of crystalline structures in the blank group, which highlights the limited impact of humic acid alone on mineral precipitation in the absence of microbial processes.

In contrast, the samples treated with 2% and 5% humic acid (Figure 3C,D) show a more pronounced presence of calcite crystals, though these are smaller and less abundant compared to the 0% humic acid group. The calcite crystals in these samples are embedded within a more compact and delicate matrix, suggesting that higher humic acid concentrations might inhibit crystal growth, possibly by interacting with carbonate ions or altering the microenvironment in a way that favours the formation of smaller crystals.

After 28 days of treatment, the samples with 1% and 10% humic acid (Figure 3E,F) displayed further changes. The 1% humic acid sample continued to show significant calcite formation, with crystals of varied sizes, while the 10% humic acid sample exhibited a smoother surface with smaller calcite crystals. The reduction in crystal size with increasing humic acid concentration aligns with findings from Zhang et al. (2019), where layered double hydroxides (LDHs) regenerated in humic acid solutions were observed to have smaller crystal sizes and reduced surface area, pore size and volume compared to those regenerated in deionized water. This suggests that humic acid may play a role in limiting crystal growth, possibly through complexation or by altering the nucleation dynamics.

Overall, the results indicate that while MICP is effective in promoting calcite formation, the presence of humic acid significantly influences the size and morphology of the precipitated minerals. The higher the concentration of humic acid, the smaller and more refined the calcite crystals, likely due to the interaction between humic acid and mineral ions in the soil. This has important implications for the use of humic acid in soil remediation, where its concentration must be carefully managed to balance microbial activity with the desired soil chemical properties.

### Chemical analyses of soil samples (by XRD and TGA)

**FIGURE 4:**
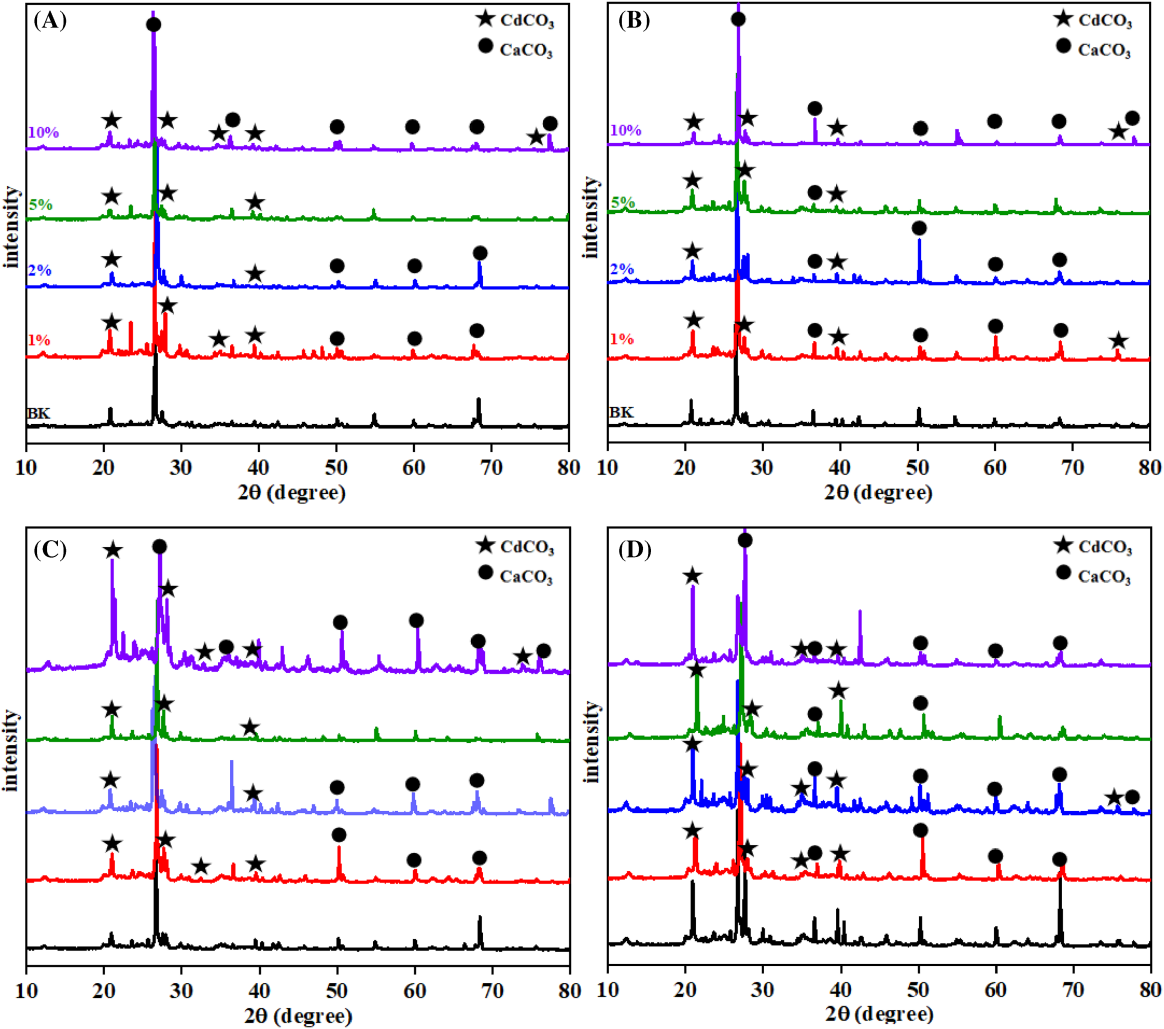
X‐ray diffraction of soils samples treated with different humic acid concentrations at (A) 7, (B) 14, (C) 21 and (D) 28 days.

To identify the biogenic minerals formed during the MICP process and to determine the primary elements involved, XRD analysis was performed on five soil samples (Figure 4). The analysis focused on detecting the presence of calcite and CdCO₃ in the treated soils, as these minerals are critical indicators of successful cadmium immobilization. The results confirmed that calcium carbonate, specifically in the form of calcite, was the predominant mineral phase resulting from the biological treatment. This finding is significant as calcite is well‐documented for its efficacy in immobilizing cadmium (Chen et al., 2023).

In the untreated soil, quartz was identified as the primary mineral component. However, following the MICP treatment, calcite emerged as the dominant mineral, indicating a successful biogenic transformation. The presence of calcite suggests that the MICP process effectively induced the precipitation of calcium carbonate, which played a key role in capturing and immobilizing cadmium within the soil matrix. Notably, the XRD analysis also detected CdCO₃, particularly in samples treated with humic acid, which increased in proportion as the concentration of humic acid rose. However, this increase in humic acid concentration corresponded with a noticeable decline in the peak intensities for both CaCO₃ and CdCO₃ in the XRD patterns, suggesting that higher levels of humic acid may inhibit carbonate formation.

**FIGURE 5:**
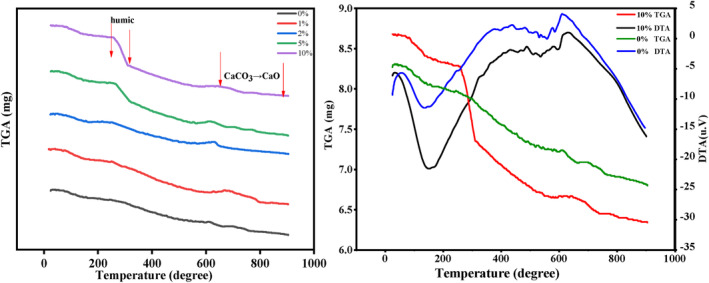
TGA‐DTA curves for humic acid‐treated soil after 28 days remediation, where TGA curves showing for various humic acid‐treated soils, while TGA‐DTA curves for untreated and 10% of humic acid‐treated soils.

The semi‐quantitative nature of XRD allowed for the identification of these biominerals, with multiple peaks indicating the presence of CdCO₃ across all experimental groups. This was further corroborated by TGA analysis (Figure 5), which provided thermal stability data for the samples. The TGA results showed a rapid weight loss between 250 and 300°C, corresponding to the decomposition of organic matter, followed by the decomposition of CdCO₃ around 400°C. The subsequent decomposition of calcium carbonate was observed between 800 and 900°C, consistent across nearly all samples. These thermal events align with previous studies (Batool et al., 2018; Li, Yang, et al., 2022), which report similar decomposition temperatures for CaCO₃ in MICP processes, typically occurring between 600 and 850°C (Polat, 2020).

The integration of XRD and TGA‐DTA data highlights the significant influence of humic acid on the MICP process. High concentrations of humic acid appear to hinder both urease activity and carbonate mineral formation, as evidenced by the reduced presence of carbonate‐bound cadmium and calcium in the treated soils. These findings suggest that while humic acid can facilitate the formation of stable organic–metal complexes, it also poses a challenge to the efficiency of the MICP process, particularly in terms of carbonate precipitation.

At the same time, however, humic acid facilitated or did not prevent formation of carbonate that can be explained based on the following factors. Humic acid contains reactive surface groups, such as carboxylic and phenolic groups, which make it highly susceptible to forming colloidal aggregates. These aggregates have the potential to act as binding sites for different contaminants, allowing the humic acid to effectively regulate their surface sorption, degradation, complexation and overall destiny. As a result, humic acid is frequently utilized in various scientific research fields, such as agriculture fertilizers and the removal of environmental pollutants (Ai et al., 2020). Incorporating humic acid amendments has also been shown to enhance soil functions, improve anti‐acidification and fertility and reduce the migration and bioavailability of heavy metal pollutants in the soil (Rong et al., 2020).

## CONCLUSION

This research evaluated the impact of humic acid on microbially induced carbonate precipitation (MICP), a technique widely used for heavy metal immobilization. The findings demonstrate that humic acid, with its porous structure, effectively absorbs heavy metals and facilitates the formation of stable organic–metal complexes. However, it also inhibits key processes in MICP, particularly urease activity and the subsequent formation of calcium carbonate and heavy metal carbonates. Specifically, as the concentration of humic acid increased, there was a significant rise in the content of organically bound heavy metals, with the 10% humic acid group showing a 32.8% increase compared to the control. Concurrently, a notable decrease in urease activity was observed, with a 23.8% reduction in the 10% humic acid group, indicating that higher concentrations of humic acid suppress urease production and activity. Additionally, analytical techniques revealed a 23.9% reduction in carbonate‐bound cadmium in the 10% humic acid group compared to the group without humic acid. These results provide valuable insights into the dual role of humic acid in MICP‐based remediation, where it both enhances heavy metal adsorption and inhibits MICP efficiency. Future research should focus on optimizing the concentration of humic acid in MICP protocols to maximize remediation effectiveness while minimizing inhibitory effects.

## AUTHOR CONTRIBUTIONS

**Wei Zhou:** Writing – original draft; software; formal analysis; data curation; investigation. **Yaqi Zhu:** Data curation; formal analysis. **Varenyam Achal:** Conceptualization; methodology; writing – review and editing; supervision.

## CONFLICT OF INTEREST STATEMENT

The authors declare no conflicts of interest.

## Data Availability

The data that support the findings of this study are available from the corresponding author upon request.
